# Supplementary material for: Socio-demographic characteristics associated with COVID-19 vaccination uptake in Switzerland: longitudinal analysis of the CoMix study
Source: BMC Public Health. 2023 Aug 10;23:1523. doi: 10.1186/s12889-023-16405-0 (PMC10413773; doi:10.1186/s12889-023-16405-0)
Supplement: Supplementary file 1 — Supplementary Material 1 [file 12889_2023_16405_MOESM1_ESM.docx]

**Supplementary material**

**For manuscript entitled:** Socio-demographic characteristics associated with COVID-19 vaccination uptake in Switzerland: longitudinal analysis of the CoMix study

**Authors:** Martina L Reichmuth^1*^, Leonie Heron^1^, Julien Riou^1,2^, André Moser^3^, Anthony Hauser^1^, Nicola Low^1,2^, Christian L Althaus^1,2^

**Affiliation**: ^1^ Institute of Social and Preventive Medicine, University of Bern, Bern, Switzerland; ^2^ Multidisciplinary Center for Infectious Diseases, University of Bern, Bern, Switzerland; ^3^ CTU Bern, University of Bern, Bern, Switzerland.

**Correspondence:** *[martina.reichmuth@unibe.ch](mailto:martina.reichmuth@unibe.ch)


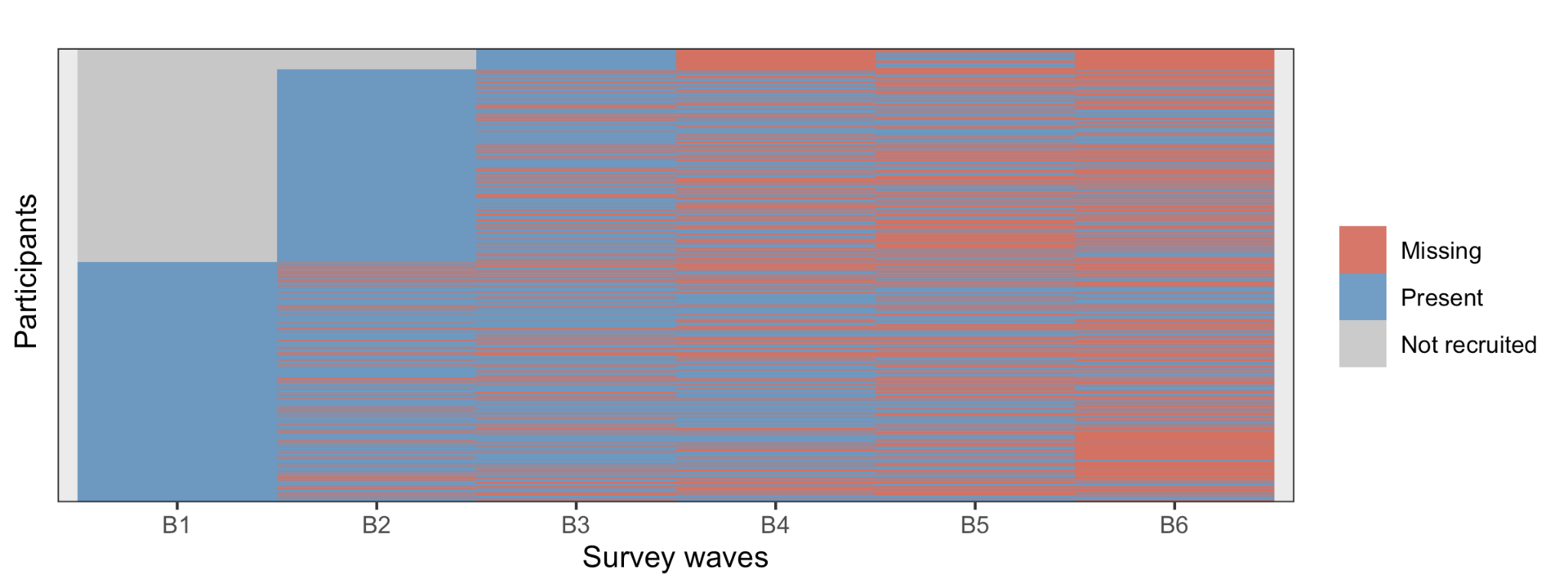


**Supplementary Figure 1:** Participants in the six survey waves of the CoMix study in Switzerland.


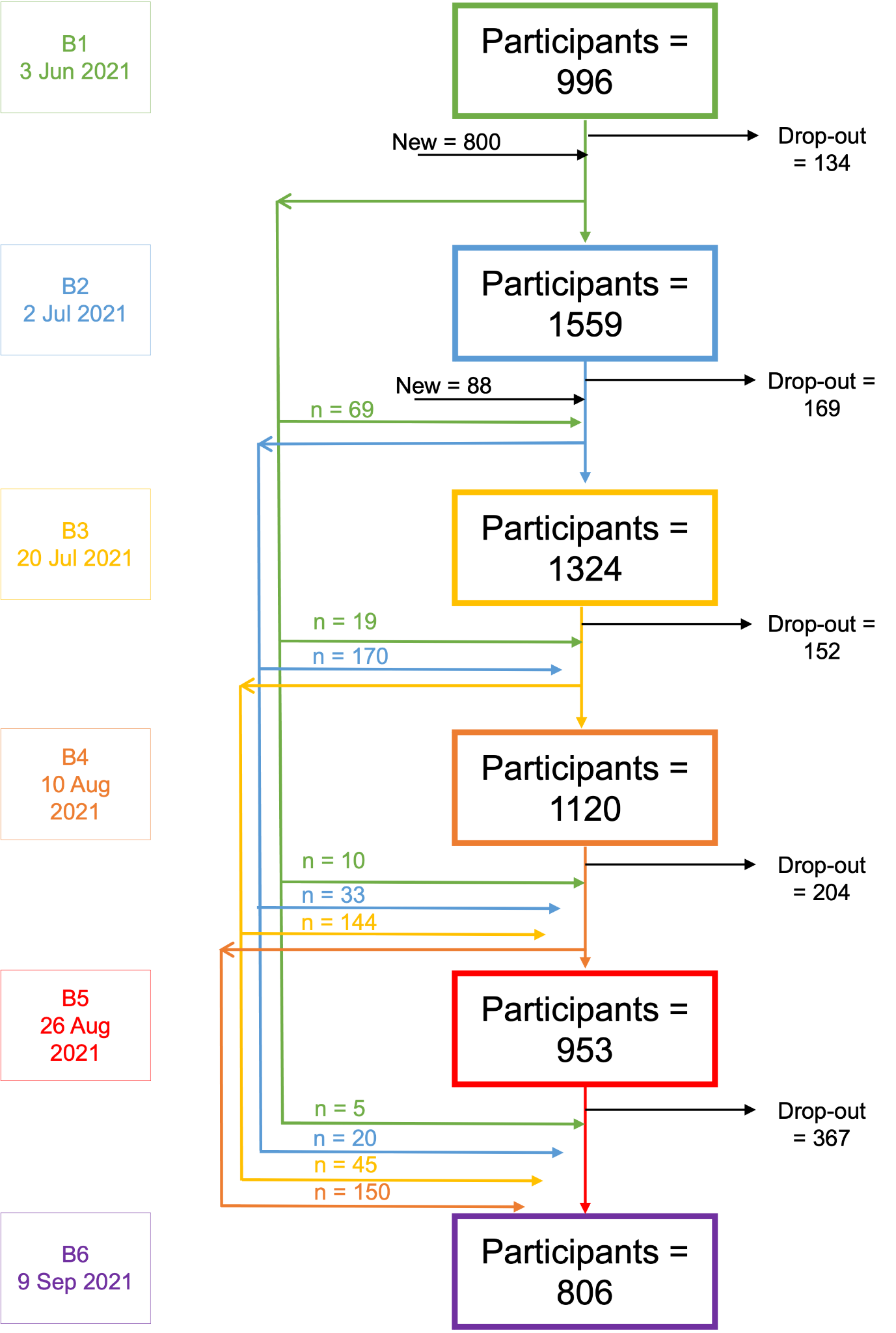


**Supplementary Figure 2**: Flow chart of the participants in the six survey waves of the CoMix study in Switzerland.


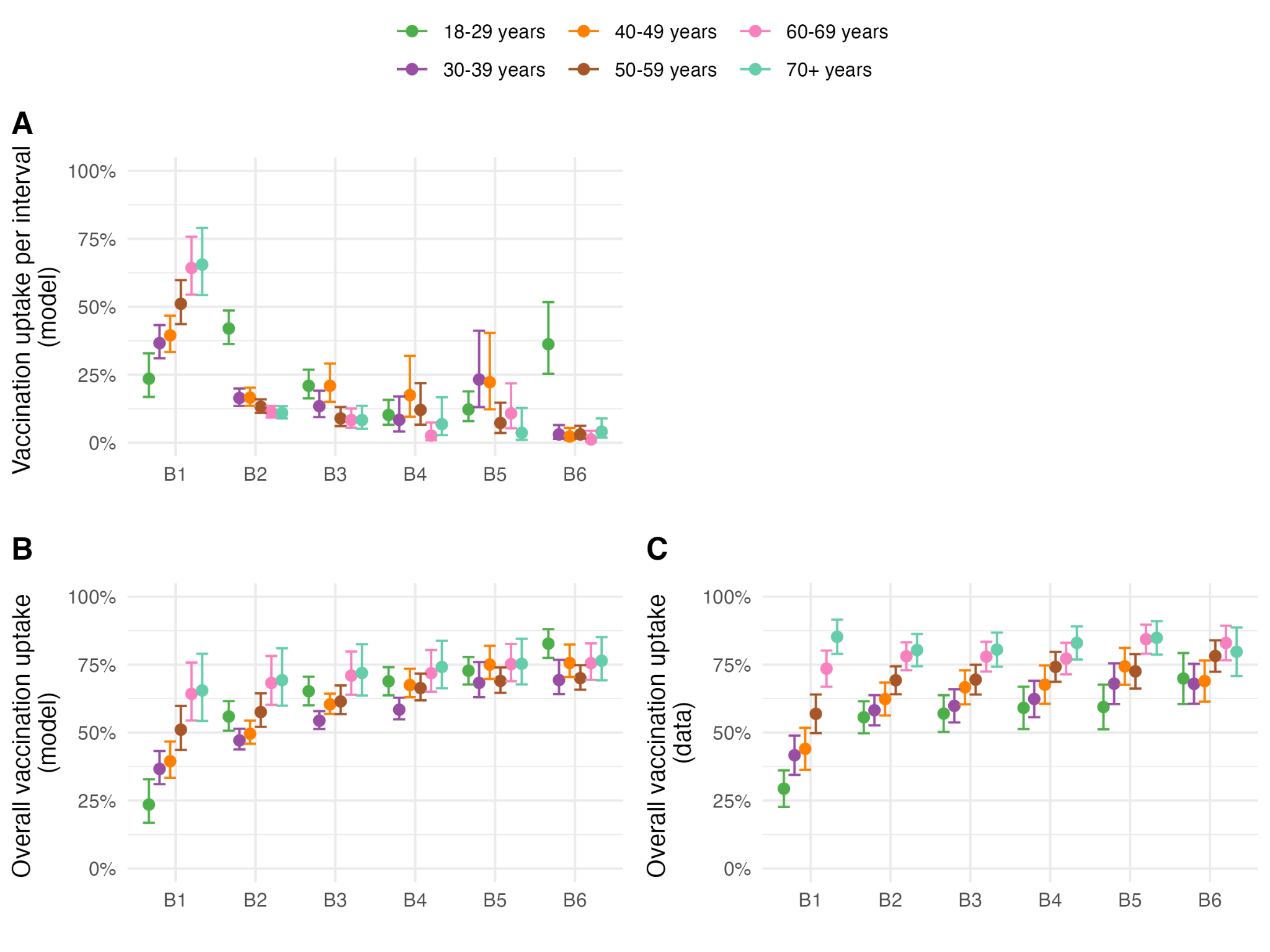


**Supplementary Figure 3:** Vaccination uptake per interval by age group. A) Vaccination uptake corresponds to the percentage receiving the first vaccine dose amongst those who have not already received it. Point estimates and confidence intervals (CIs) as predicted by the Poisson regression model. The estimates were adjusted for gender, region, Swiss region of residence, country of birth, education level, employment level, net household income, household size, household with a medically vulnerable individual, testing for SARS-CoV-2, number of contacts per day, and attitude towards COVID-19 measures, with an interaction between age and survey wave. B) Cumulative (overall) vaccination uptake corresponds to the percentage of having received vaccination as predicted by the Poisson regression model. CIs were derived from multivariate parameter samples using the covariance matrix of model estimates C) Overall vaccination uptake of the study population per interval. Point estimates and binomial CIs were calculated separately for each survey wave from the raw data. The error bars indicate the 95% CIs.

**Supplementary Table 1:** Milestones of the vaccination program in Switzerland. * Varied across cantons.

| **Dates** | **Description (eligible subpopulation)** | **Reference** accessed on 17 May 2023, in German |
| --- | --- | --- |
| 19 Dec 2020 | Swissmedic, the Swiss agency for the authorisation and supervision of therapeutic products, authorised the mRNA vaccine from Pfizer/BioNTech. | https://www.bag.admin.ch/bag/de/home/das-bag/aktuell/medienmitteilungen.msg-id-81667.html |
| 23 Dec 2020 | First person officially received the first vaccine dose in Switzerland. | https://www.srf.ch/news/schweiz/impfstart-in-der-schweiz-luzernerin-erhaelt-erste-impfung-auch-weitere-kantone-gestartet |
| 24 Dec 2020 | Switzerland started the COVID-19 vaccination campaign. Priority was given to the elderly (>75 and then 65-75 years) and the chronically ill, and, secondly*, to healthcare workers and those living with people at risk. | https://www.bag.admin.ch/bag/de/home/das-bag/aktuell/medienmitteilungen.msg-id-81798.html |
| 12 Jan 2021 | Swissmedic authorised the mRNA vaccine from Moderna. | https://www.bag.admin.ch/bag/de/home/das-bag/aktuell/medienmitteilungen.msg-id-81926.html |
| May 2021* | General population (≥16 years) was able to get vaccinated. |  |
| 31 Mai 2021 | Vaccinated people were exempt from quarantine, including quarantine after returning from abroad. | https://www.bag.admin.ch/bag/de/home/das-bag/aktuell/medienmitteilungen.msg-id-83531.html |
| June 2021 | Children (≥12 years) were able to get vaccinated. | https://www.bag.admin.ch/bag/de/home/das-bag/aktuell/medienmitteilungen.msg-id-84095.html |
| 13 Sep 2021 | COVID-19 certificate introduced in September 2021: proof of vaccination, recovery or a negative test result were declared mandatory to access indoor hospitality venues, cultural, sporting and leisure activities indoors, and large-scale outdoor events. | https://www.bag.admin.ch/bag/de/home/das-bag/aktuell/medienmitteilungen.msg-id-85035.html |
| 1 Jan 2022 | Children (≥5 years) were able to get vaccinated. | https://www.bag.admin.ch/bag/de/home/das-bag/aktuell/medienmitteilungen.msg-id-86451.html |

**Supplementary Table 2:** Comparison of key characteristics of study participants with the Swiss population. The income of study participants is the household income, whereas the income for the Swiss population corresponds to the individual income from full and part-time employees.

| **Category** | **Name** | **Swiss population, n (%)** | **Study participants, n (%)** | **Survey wave, n (%)** | | | | | |
| --- | --- | --- | --- | --- | --- | --- | --- | --- | --- |
|  |  |  |  | **B1** | **B2** | **B3** | **B4** | **B5** | **B6** |
| Gender | Female | 4,367,701 (50.4%) | 3,297 (48.8%) | 497 (49.9%) | 761 (48.8%) | 645 (48.7%) | 556 (49.6%) | 452 (47.4%) | 386 (47.9%) |
|  | Male | 4,302,599 (49.6%) | 3,436 (50.8%) | 495 (49.7%) | 792 (50.8%) | 673 (50.8%) | 560 (50%) | 498 (52.3%) | 418 (51.9%) |
|  | Other | - | 25 (0.4%) | 4 (0.4%) | 6 (0.4%) | 6 (0.5%) | 4 (0.4%) | 3 (0.3%) | 2 (0.2%) |
| Age group | 18-29 | 1,170,597 (16.3%) | 1,044 (15.4%) | 177 (17.8%) | 275 (17.6%) | 207 (15.6%) | 154 (13.8%) | 138 (14.5%) | 93 (11.5%) |
|  | 30-39 | 1,239,355 (17.2%) | 1,241 (18.4%) | 180 (18.1%) | 304 (19.5%) | 249 (18.8%) | 202 (18%) | 150 (15.7%) | 156 (19.4%) |
|  | 40-49 | 1,200,424 (16.7%) | 1,100 (16.3%) | 159 (16%) | 247 (15.8%) | 219 (16.5%) | 170 (15.2%) | 160 (16.8%) | 145 (18%) |
|  | 50-59 | 1,304,794 (18.1%) | 1,407 (20.8%) | 188 (18.9%) | 309 (19.8%) | 273 (20.6%) | 245 (21.9%) | 194 (20.4%) | 198 (24.6%) |
|  | 60-69 | 1,005,687 (14%) | 1,159 (17.2%) | 170 (17.1%) | 251 (16.1%) | 222 (16.8%) | 202 (18%) | 179 (18.8%) | 135 (16.7%) |
|  | 70+ | 1,276,149 (17.7%) | 807 (11.9%) | 122 (12.2%) | 173 (11.1%) | 154 (11.6%) | 147 (13.1%) | 132 (13.9%) | 79 (9.8%) |
| Household income* | 0-5,000 | 49.4% | 2,150 (31.8%) | 328 (32.9%) | 493 (31.6%) | 425 (32.1%) | 359 (32.1%) | 309 (32.4%) | 236 (29.3%) |
|  | 5,001-10,000 | 42.2% | 2,733 (40.4%) | 396 (39.8%) | 634 (40.7%) | 533 (40.3%) | 445 (39.7%) | 384 (40.3%) | 341 (42.3%) |
|  | 10,000+ | 7.6% | 861 (12.7%) | 124 (12.4%) | 203 (13%) | 171 (12.9%) | 139 (12.4%) | 122 (12.8%) | 102 (12.7%) |
|  | Preferred not to answer | - | 1,014 (15%) | 148 (14.9%) | 229 (14.7%) | 195 (14.7%) | 177 (15.8%) | 138 (14.5%) | 127 (15.8%) |
| Residence | Espace Mittelland | 1,895,693 (21.9%) | 1,489 (22%) | 216 (21.7%) | 344 (22.1%) | 286 (21.6%) | 241 (21.5%) | 226 (23.7%) | 176 (21.8%) |
|  | Zurich | 1,553,423 (17.9%) | 1,287 (19%) | 188 (18.9%) | 291 (18.7%) | 244 (18.4%) | 209 (18.7%) | 190 (19.9%) | 165 (20.5%) |
|  | Lake Geneva region | 1,669,608 (19.3%) | 1,126 (16.7%) | 168 (16.9%) | 273 (17.5%) | 225 (17%) | 191 (17.1%) | 165 (17.3%) | 104 (12.9%) |
|  | Eastern Switzerland | 1,193,069 (13.8%) | 963 (14.2%) | 141 (14.2%) | 219 (14%) | 192 (14.5%) | 161 (14.4%) | 138 (14.5%) | 112 (13.9%) |
|  | Northwestern Switzerland | 1,181,776 (13.6%) | 949 (14%) | 143 (14.4%) | 219 (14%) | 186 (14%) | 155 (13.8%) | 132 (13.9%) | 114 (14.1%) |
|  | Central Switzerland | 825,745 (9.5%) | 678 (10%) | 96 (9.6%) | 148 (9.5%) | 137 (10.3%) | 108 (9.6%) | 97 (10.2%) | 92 (11.4%) |
|  | Ticino | 350,986 (4%) | 266 (3.9%) | 44 (4.4%) | 65 (4.2%) | 54 (4.1%) | 55 (4.9%) | 5 (0.5%) | 43 (5.3%) |

*No absolute values available for net income of the Swiss population.

**Supplementary Table 3:** Comparison of different models to study the association of socio-demographic and other factors with COVID-19 vaccination uptake in Switzerland. Abbreviations: CI, confidence interval; HR, hazard ratio; RR, rate ratio.

| **Name** | **Categories** | **Number of participants** | **Number of answers** | **Cox proportional hazard model** | | | | **Poisson regression model** | | | | |
| --- | --- | --- | --- | --- | --- | --- | --- | --- | --- | --- | --- | --- |
|  | | | | **Unadjusted HR (95% CI)** | **Unadjusted weighted HR (95% CI)** | **Adjusted HR (95% CI)** | **Adjusted weighted HR (95% CI)** | **Unadjusted RR (95% CI)** | **Unadjusted RR (95% CI) with age as interaction** | **Adjusted RR (95% CI)** | **Adjusted RR (95% CI) with age as interaction** | **Adjusted RR (95% CI) with age as interaction (1 June)** |
| Survey wave  Reference: B1 | B2 | 1,143 | - | - | - | - | - | 1.07 (1.01-1.13) | - | - | - | - |
|  | B3 | 511 | - | - | - | - | - | 0.43 (0.39-0.48) | - | - | - | - |
|  | B4 | 356 | - | - | - | - | - | 0.15 (0.13-0.19) | - | - | - | - |
|  | B5 | 277 | - | - | - | - | - | 0.23 (0.19-0.28) | - | - | - | - |
|  | B6 | 230 | - | - | - | - | - | 0.20 (0.16-0.25) | - | - | - | - |
| Age groups, years  Reference: 18-29 | 30-39 | 737 | 359 | 1.05 (0.87-1.26) | 1.15 (0.98-1.36) | - | - | 1.07 (0.98-1.16) | - | - | - | - |
|  | 40-49 | 606 | 314 | 1.09 (0.90-1.32) | 1.15 (0.97-1.36) | - | - | 1.17 (1.07-1.27) | - | - | - | - |
|  | 50-59 | 676 | 355 | 1.26 (1.05-1.51) | 1.29 (1.10-1.52) | - | - | 1.30 (1.20-1.42) | - | - | - | - |
|  | 60-69 | 474 | 289 | 1.67 (1.39-2.01) | 1.81 (1.51-2.18) | - | - | 1.62 (1.49-1.77) | - | - | - | - |
|  | 70+ | 318 | 202 | 2.10 (1.72-2.56) | 2.18 (1.79-2.66) | - | - | 1.85 (1.69-2.03) | - | - | - | - |
| Gender  Reference: Female | Male | 1,708 | 955 | 1.17 (1.05-1.30) | 1.05 (0.95-1.16) | 1.07 (0.95-1.20) | 0.96 (0.86-1.07) | 1.17 (1.11-1.23) | 1.12 (1.06-1.17) | 1.09 (1.04-1.15) | 1.09 (1.04-1.15) | 1.11 (1.04-1.19) |
|  | Others | 15 | 10 | 1.24 (0.64-2.39) | 0.95 (0.68-1.33) | 0.86 (0.44-1.69) | 0.99 (0.56-1.75) | 1.62 (1.20-2.17) | 1.76 (1.31-2.38) | 1.51 (1.12-2.04) | 1.62 (1.20-2.20) | 1.78 (1.23-2.57) |
| Region  Reference: Urban | Rural | 961 | 456 | 0.75 (0.66-0.86) | 0.80 (0.71-0.90) | 0.89 (0.77-1.02) | 0.86 (0.76-0.97) | 0.75 (0.71-0.80) | 0.79 (0.74-0.84) | 0.84 (0.79-0.90) | 0.85 (0.80-0.90) | 0.85 (0.78-0.92) |
| Swiss regions of residence  Reference: Espace Mittelland | Zurich | 615 | 351 | 1.22 (1.03-1.44) | 1.14 (0.99-1.33) | 1.23 (1.03-1.47) | 1.11 (0.94-1.30) | 1.23 (1.14-1.33) | 1.19 (1.10-1.29) | 1.10 (1.02-1.20) | 1.11 (1.02-1.20) | 1.12 (1.01-1.25) |
|  | Lake Geneva region | 590 | 337 | 1.12 (0.94-1.33) | 1.06 (0.92-1.23) | 1.02 (0.86-1.22) | 1.02 (0.88-1.19) | 1.17 (1.08-1.27) | 1.13 (1.05-1.23) | 1.06 (0.98-1.15) | 1.06 (0.98-1.15) | 1.05 (0.95-1.16) |
|  | Eastern Switzerland | 501 | 263 | 1.09 (0.91-1.32) | 1.03 (0.88-1.21) | 1.14 (0.95-1.38) | 1.10 (0.93-1.30) | 1.10 (1.01-1.20) | 1.09 (1.00-1.19) | 1.10 (1.01-1.20) | 1.09 (1.00-1.18) | 1.09 (0.98-1.22) |
|  | Northwestern Switzerland | 262 | 262 | 1.11 (0.92-1.34) | 1.07 (0.91-1.26) | 1.28 (1.05-1.55) | 1.17 (0.99-1.39) | 1.07 (0.98-1.16) | 1.08 (0.99-1.17) | 1.08 (0.99-1.18) | 1.07 (0.98-1.17) | 1.09 (0.97-1.22) |
|  | Central Switzerland | 352 | 182 | 1.07 (0.87-1.32) | 0.97 (0.80-1.17) | 1.23 (0.99-1.52) | 1.06 (0.86-1.31) | 1.05 (0.95-1.16) | 1.11 (1.01-1.23) | 1.18 (1.07-1.30) | 1.16 (1.05-1.28) | 1.20 (1.06-1.36) |
|  | Ticino | 140 | 82 | 1.29 (0.97-1.72) | 1.59 (1.11-2.27) | 1.50 (1.12-2.02) | 1.74 (1.20-2.53) | 1.16 (1.02-1.32) | 1.16 (1.02-1.33) | 1.14 (1.00-1.30) | 1.15 (1.01-1.31) | 1.18 (1.00-1.40) |
| Country of birth  Reference: Switzerland | EU | 450 | 249 | 1.10 (0.93-1.29) | 1.06 (0.93-1.22) | 1.09 (0.92-1.29) | 1.09 (0.94-1.27) | 1.05 (0.98-1.13) | 1.02 (0.94-1.10) | 0.97 (0.90-1.04) | 0.96 (0.89-1.04) | 0.97 (0.88-1.07) |
|  | Non-EU | 283 | 156 | 1.11 (0.91-1.35) | 1.13 (0.96-1.33) | 1.03 (0.84-1.25) | 1.08 (0.89-1.30) | 1.06 (0.97-1.16) | 1.01 (0.93-1.11) | 0.96 (0.88-1.05) | 0.98 (0.89-1.07) | 1.01 (0.90-1.13) |
|  | Unknown | 235 | 147 | 1.18 (0.97-1.45) | 1.11 (0.86-1.44) | 1.10 (0.89-1.36) | 1.07 (0.83-1.38) | 1.18 (1.08-1.29) | 1.03 (0.94-1.13) | 1.06 (0.97-1.16) | 1.06 (0.97-1.17) | 1.10 (0.97-1.24) |
| Education level  Reference: Lowest level | Middle level of education | 1,225 | 639 | 1.08 (0.95-1.22) | 1.11 (0.99-1.25) | 1.00 (0.88-1.14) | 1.04 (0.92-1.17) | 1.06 (1.00-1.12) | 1.06 (1.00-1.13) | 1.01 (0.95-1.08) | 1.01 (0.95-1.07) | 0.99 (0.92-1.07) |
|  | Highest level of education | 714 | 439 | 1.41 (1.23-1.61) | 1.23 (1.10-1.37) | 1.26 (1.08-1.46) | 1.17 (1.02-1.33) | 1.39 (1.31-1.48) | 1.35 (1.27-1.43) | 1.18 (1.10-1.26) | 1.18 (1.10-1.27) | 1.19 (1.09-1.30) |
| Employment status  Reference: Employed | Unemployed | 214 | 110 | 0.88 (0.69-1.13) | 1.04 (0.77-1.40) | 0.89 (0.69-1.15) | 1.04 (0.77-1.42) | 0.86 (0.77-0.96) | 0.83 (0.74-0.93) | 0.86 (0.77-0.97) | 0.86 (0.76-0.97) | 0.88 (0.76-1.03) |
|  | Student | 192 | 116 | 1.23 (0.98-1.54) | 1.04 (0.86-1.25) | 1.55 (1.17-2.05) | 1.27 (0.98-1.64) | 1.13 (1.02-1.26) | 1.38 (1.22-1.56) | 1.26 (1.11-1.43) | 1.33 (1.17-1.51) | 1.35 (1.15-1.58) |
|  | Homemaker | 164 | 75 | 0.73 (0.54-0.99) | 0.75 (0.58-0.97) | 0.95 (0.69-1.32) | 0.81 (0.63-1.05) | 0.77 (0.67-0.89) | 0.80 (0.70-0.92) | 0.96 (0.83-1.10) | 0.95 (0.82-1.10) | 0.97 (0.81-1.16) |
|  | Retired | 607 | 377 | 1.66 (1.45-1.89) | 1.63 (1.44-1.85) | 1.20 (0.95-1.51) | 1.11 (0.89-1.38) | 1.46 (1.38-1.55) | 1.01 (0.91-1.12) | 1.05 (0.95-1.17) | 1.05 (0.94-1.16) | 1.08 (0.94-1.24) |
|  | Other unemployed situation | 92 | 44 | 0.85 (0.58-1.26) | 1.07 (0.78-1.47) | 0.96 (0.64-1.44) | 1.30 (0.91-1.85) | 0.85 (0.71-1.01) | 0.82 (0.69-0.98) | 0.92 (0.77-1.10) | 0.90 (0.75-1.07) | 0.80 (0.63-1.03) |
| Household income, net  Reference: 0-5,000 CHF | 5,001-10,000 CHF | 1,403 | 762 | 1.19 (1.04-1.36) | 1.14 (1.01-1.28) | 1.25 (1.08-1.44) | 1.24 (1.08-1.42) | 1.18 (1.11-1.25) | 1.21 (1.14-1.28) | 1.15 (1.08-1.23) | 1.15 (1.08-1.23) | 1.14 (1.04-1.24) |
|  | 10,000+ CHF | 387 | 248 | 1.49 (1.26-1.77) | 1.25 (1.08-1.45) | 1.42 (1.17-1.73) | 1.29 (1.09-1.54) | 1.50 (1.39-1.62) | 1.47 (1.36-1.59) | 1.33 (1.21-1.45) | 1.34 (1.23-1.46) | 1.36 (1.22-1.53) |
|  | Preferred not to answer | 529 | 281 | 1.16 (0.98-1.38) | 1.17 (1.00-1.37) | 1.14 (0.95-1.36) | 1.15 (0.98-1.36) | 1.17 (1.08-1.27) | 1.16 (1.07-1.25) | 1.14 (1.06-1.24) | 1.13 (1.04-1.23) | 1.15 (1.03-1.27) |
| Household size | Mean (range) | 2 (1 - 10) | 2 (1 - 10) | 0.96 (0.92-1.00) | 0.95 (0.91-0.98) | 0.95 (0.89-1.01) | 0.97 (0.92-1.02) | 0.97 (0.95-0.98) | 1.02 (0.99-1.04) | 0.96 (0.94-0.99) | 0.96 (0.94-0.99) | 0.97 (0.94-1.01) |
| Household with medically vulnerability  Reference: No person in a risk group | One or more person in a risk group | 944 | 578 | 1.33 (1.19-1.49) | 1.23 (1.11-1.37) | 1.21 (1.07-1.36) | 1.18 (1.05-1.33) | 1.36 (1.30-1.44) | 1.20 (1.13-1.26) | 1.18 (1.11-1.24) | 1.16 (1.10-1.23) | 1.16 (1.08-1.25) |
| Testing for SARS-CoV-2  Reference: Tested positive | Tested | 872 | - | 0.85 (0.46-1.55) | 1.11 (0.73-1.70) | 0.98 (0.53-1.82) | 1.23 (0.77-1.95) | 0.73 (0.55-0.98) | 0.75 (0.56-1.00) | 0.85 (0.63-1.13) | 0.87 (0.65-1.17) | 0.76 (0.55-1.06) |
|  | Never tested | 2,557 | - | 1.72 (0.95-3.11) | 1.72 (1.14-2.58) | 1.96 (1.07-3.59) | 1.84 (1.17-2.90) | 1.09 (0.82-1.44) | 0.87 (0.65-1.15) | 1.02 (0.77-1.37) | 1.06 (0.80-1.42) | 0.96 (0.69-1.32) |
|  | Preferred not to answer | 56 | - | 0.96 (0.43-2.15) | 1.13 (0.55-2.31) | 1.59 (0.70-3.61) | 1.53 (0.70-3.35) | 0.58 (0.40-0.85) | 0.55 (0.38-0.81) | 0.74 (0.50-1.09) | 0.76 (0.51-1.12) | 0.61 (0.38-0.98) |
| Number of contacts per day  Reference: 0-2 | 3-5 | 937 | - | 1.13 (0.98-1.30) | 1.08 (0.95-1.22) | 1.23 (1.07-1.43) | 1.13 (0.99-1.29) | 1.03 (0.97-1.09) | 1.02 (0.96-1.09) | 1.02 (0.95-1.09) | 1.02 (0.95-1.09) | 1.01 (0.92-1.10) |
|  | 6+ | 1,282 | - | 1.16 (1.02-1.31) | 1.01 (0.90-1.13) | 1.28 (1.10-1.49) | 1.13 (0.98-1.30) | 1.06 (1.00-1.12) | 1.09 (1.03-1.16) | 1.08 (1.01-1.16) | 1.08 (1.01-1.16) | 1.10 (1.01-1.20) |
| Attitudes towards COVID-19 measures  Reference: About right | Too lenient | 663 | - | 0.88 (0.77-1.01) | 0.81 (0.73-0.90) | 0.85 (0.74-0.97) | 0.79 (0.70-0.88) | 1.02 (0.96-1.08) | 1.05 (0.99-1.12) | 1.01 (0.95-1.07) | 1.02 (0.96-1.08) | 1.03 (0.95-1.11) |
|  | Too strict | 1,157 | - | 0.32 (0.28-0.37) | 0.49 (0.41-0.58) | 0.33 (0.28-0.39) | 0.51 (0.42-0.60) | 0.44 (0.41-0.47) | 0.55 (0.52-0.59) | 0.56 (0.52-0.60) | 0.56 (0.53-0.61) | 0.52 (0.48-0.57) |
|  | Don't know | 111 | - | 0.43 (0.29-0.63) | 0.53 (0.38-0.74) | 0.45 (0.31-0.68) | 0.56 (0.39-0.80) | 0.51 (0.43-0.61) | 0.61 (0.51-0.72) | 0.63 (0.53-0.76) | 0.64 (0.53-0.77) | 0.70 (0.56-0.88) |

**Supplementary Table 4:** Cox proportional hazard model to study the association of socio-demographic and other factors with COVID-19 vaccination uptake in Switzerland. Compared to Supplementary Table 3, Supplementary Table 4 shows participants that either got not vaccinated during our study or had an exact date of vaccination. Abbreviations: CI, confidence interval; HR, hazard ratio.

| **Name** | **Categories** | **Number of answers** | **Unadjusted HR (95% CI)** | **Adjusted weighted HR (95% CI)** |
| --- | --- | --- | --- | --- |
| Age groups, years  Reference: 18-29 | 30-39 | 336 | 1.31 (1.07-1.61) |  |
|  | 40-49 | 282 | 1.31 (1.06-1.62) |  |
|  | 50-59 | 334 | 1.89 (1.55-2.31) |  |
|  | 60-69 | 266 | 2.98 (2.42-3.66) |  |
|  | 70+ | 186 | 4.78 (3.83-5.97) |  |
| Gender  Reference: Female | Male | 869 | 1.05 (0.93-1.17) | 0.95 (0.84-1.07) |
|  | Others | 7 | 2.29 (0.95-5.53) | 2.23 (0.90-5.56) |
| Region  Reference: Urban | Rural | 430 | 0.89 (0.78-1.03) | 0.98 (0.85-1.15) |
| Swiss regions of residence  Reference: Espace Mittelland | Zurich | 323 | 1.12 (0.93-1.34) | 0.98 (0.82-1.18) |
|  | Lake Geneva region | 316 | 1.12 (0.93-1.35) | 1.09 (0.90-1.32) |
|  | Eastern Switzerland | 247 | 1.10 (0.90-1.34) | 1.05 (0.86-1.27) |
|  | Northwestern Switzerland | 231 | 1.08 (0.87-1.33) | 1.13 (0.91-1.39) |
|  | Central Switzerland | 161 | 0.87 (0.69-1.09) | 0.95 (0.76-1.20) |
|  | Ticino | 77 | 1.01 (0.74-1.36) | 1.24 (0.83-1.85) |
| Country of birth  Reference: Switzerland | EU | 237 | 0.99 (0.84-1.17) | 0.95 (0.80-1.12) |
|  | Non-EU | 150 | 0.95 (0.77-1.17) | 0.93 (0.76-1.14) |
|  | Unknown | 117 | 1.10 (0.87-1.40) | 1.08 (0.82-1.41) |
| Education level  Reference: Lowest level | Middle level of education | 584 | 1.01 (0.88-1.16) | 1.17 (1.01-1.35) |
|  | Highest level of education | 397 | 1.02 (0.89-1.19) | 1.26 (1.08-1.47) |
| Employment status  Reference: Employed | Unemployed | 104 | 1.01 (0.77-1.31) | 1.03 (0.76-1.39) |
|  | Student | 97 | 0.71 (0.55-0.92) | 0.95 (0.70-1.27) |
|  | Homemaker | 68 | 0.99 (0.70-1.40) | 1.00 (0.72-1.38) |
|  | Retired | 348 | 2.47 (2.15-2.85) | 1.24 (0.98-1.57) |
|  | Other unemployed situation | 41 | 1.83 (1.21-2.78) | 1.56 (1.06-2.29) |
| Household income, net  Reference: 0-5,000 CHF | 5,001-10,000 CHF | 704 | 0.95 (0.83-1.10) | 1.36 (1.16-1.60) |
|  | 10,000+ CHF | 231 | 0.90 (0.75-1.08) | 1.26 (1.01-1.56) |
|  | Preferred not to answer | 238 | 0.91 (0.75-1.11) | 1.02 (0.84-1.24) |
| Household size | Mean (range) | 2 (1 - 9) | 0.85 (0.81-0.90) | 0.88 (0.82-0.94) |
| Household with medically vulnerability  Reference: No person in a risk group | One or more person in a risk group | 529 | 1.46 (1.29-1.65) | 1.50 (1.31-1.72) |
| Testing for SARS-CoV-2  Reference: Tested positive | Tested | 316 | 0.81 (0.40-1.63) | 0.75 (0.33-1.73) |
|  | Never Tested | 1376 | 1.37 (0.68-2.75) | 1.06 (0.46-2.41) |
|  | Preferred not to answer | 25 | 0.56 (0.21-1.49) | 0.56 (0.19-1.64) |
| Number of contacts per day  Reference: 0-2 | 3-5 | 462 | 1.01 (0.87-1.17) | 1.17 (1.01-1.37) |
|  | 6+ | 692 | 0.87 (0.76-0.99) | 1.11 (0.94-1.30) |
| Attitudes towards COVID-19 measures  Reference: About right | Too lenient | 368 | 0.94 (0.82-1.08) | 0.84 (0.73-0.96) |
|  | Too strict | 420 | 0.74 (0.62-0.87) | 0.82 (0.69-0.97) |
|  | Don't know | 46 | 0.39 (0.24-0.62) | 0.72 (0.36-1.44) |

**Supplementary Table 5:** Results from the logistic regression model to study the association of study participants’ characteristics with missed survey waves. Abbreviations: CI, confidence interval; OR, odds ratio; adjusted OR, aOR.

| **Category** | **Variables** | **Number of participants** | **Number of observations** | **Univariable OR (95% CI)** | **aOR (95% CI) without time varying variables** | **aOR (95% CI) with time varying variables** |
| --- | --- | --- | --- | --- | --- | --- |
| Age groups, years  Reference: 18-29 | 30-39 | 358 | 358 | 0.54 (0.36-0.81) | 0.94 (0.88-1.00) | 0.93 (0.87-1.00) |
|  | 40-49 | 308 | 308 | 0.41 (0.27-0.61) | 0.90 (0.83-0.96) | 0.89 (0.83-0.95) |
|  | 50-59 | 363 | 363 | 0.28 (0.19-0.41) | 0.84 (0.78-0.90) | 0.84 (0.78-0.90) |
|  | 60-69 | 289 | 289 | 0.35 (0.23-0.52) | 0.90 (0.82-0.98) | 0.90 (0.82-0.98) |
|  | 70+ | 207 | 207 | 0.37 (0.24-0.57) | 0.91 (0.82-1.02) | 0.92 (0.82-1.03) |
| Gender  Reference: Female | Male | 955 | 955 | 0.91 (0.74-1.13) | 0.98 (0.94-1.02) | 0.98 (0.95-1.02) |
|  | Others | 10 | 10 | 1.17 (0.25-5.56) | 1.03 (0.79-1.34) | 1.04 (0.80-1.34) |
| Region  Reference: Urban | Rural | 457 | 457 | 0.86 (0.67-1.09) | 0.99 (0.94-1.04) | 0.99 (0.94-1.03) |
| Swiss region of residence  Reference: Espace Mittelland | Zurich | 351 | 351 | 0.97 (0.70-1.34) | 0.99 (0.93-1.05) | 0.99 (0.93-1.05) |
|  | Lake Geneva region | 337 | 337 | 1.66 (1.16-2.38) | 1.08 (1.01-1.14) | 1.07 (1.01-1.13) |
|  | Eastern Switzerland | 263 | 263 | 1.08 (0.75-1.54) | 1.01 (0.94-1.07) | 1.01 (0.94-1.07) |
|  | Northwestern Switzerland | 262 | 262 | 1.16 (0.81-1.67) | 1.02 (0.95-1.09) | 1.02 (0.95-1.09) |
|  | Central Switzerland | 182 | 182 | 0.88 (0.60-1.31) | 0.98 (0.91-1.06) | 0.99 (0.92-1.06) |
|  | Ticino | 82 | 82 | 5.44 (2.14-13.81) | 1.22 (1.10-1.35) | 1.21 (1.10-1.34) |
| Country of birth  Reference: Switzerland | EU | 249 | 249 | 1.61 (1.13-2.30) | 1.09 (1.03-1.16) | 1.10 (1.03-1.16) |
|  | Non-EU | 156 | 156 | 1.32 (0.87-1.99) | 1.05 (0.98-1.12) | 1.05 (0.98-1.12) |
|  | Unknown | 147 | 147 | 0.85 (0.58-1.24) | 0.99 (0.92-1.06) | 0.98 (0.91-1.06) |
| Education level  Reference: Lowest level | Middle level of education | 639 | 639 | 1.03 (0.81-1.31) | 0.98 (0.94-1.03) | 0.97 (0.93-1.02) |
|  | Highest level of education | 439 | 439 | 1.18 (0.89-1.56) | 0.99 (0.93-1.04) | 0.99 (0.94-1.04) |
| Employment status  Reference: Employed | Unemployed | 110 | 110 | 1.05 (0.66-1.67) | 1.01 (0.92-1.09) | 1.00 (0.92-1.09) |
|  | Student | 116 | 116 | 5.64 (2.45-12.96) | 1.08 (0.99-1.19) | 1.09 (0.99-1.20) |
|  | Homemaker | 75 | 75 | 0.70 (0.42-1.16) | 0.92 (0.83-1.02) | 0.92 (0.83-1.02) |
|  | Retired | 377 | 377 | 0.83 (0.64-1.08) | 1.00 (0.92-1.08) | 1.00 (0.92-1.09) |
|  | Other unemployed situation | 44 | 44 | 0.73 (0.38-1.42) | 0.98 (0.86-1.12) | 0.97 (0.86-1.11) |
| Household income, net  Reference: 0-5,000 CHF | 5,001-10,000 CHF | 762 | 762 | 0.98 (0.76-1.27) | 0.98 (0.93-1.03) | 0.98 (0.93-1.02) |
|  | 10,000+ CHF | 248 | 248 | 1.07 (0.75-1.52) | 0.98 (0.91-1.05) | 0.99 (0.93-1.06) |
|  | Preferred not to answer | 281 | 281 | 0.93 (0.67-1.30) | 0.97 (0.91-1.03) | 0.98 (0.92-1.04) |
| Household size | Mean (range) | 2 (1 - 10) | 2 (1 - 10) | 1.20 (1.09-1.32) | 1.02 (1.01-1.04) | 0.99 (0.98-1.01) |
| Reference: Nonvulnerable population | Medically vulnerable population | 578 | 578 | 0.84 (0.67-1.06) | 0.97 (0.93-1.02) | 0.97 (0.93-1.02) |
| Vaccination status  Reference: Not vaccinated | Vaccinated | - | 1,321 | 0.74 (0.58-0.94) | - | 0.95 (0.91-0.99) |
| Testing for SARS-CoV-2  Reference: Tested positive | Tested | - | 543 | 0.66 (0.25-1.76) | - | 0.94 (0.81-1.09) |
|  | Never tested | - | 1,277 | 0.60 (0.23-1.58) | - | 0.95 (0.81-1.10) |
|  | Preferred not to answer | - | 32 | 0.83 (0.23-3.07) | - | 0.98 (0.79-1.21) |
| Number of contacts per day  Reference: 0-2 | 3-5 | - | 527 | 1.12 (0.87-1.43) | - | 1.02 (0.98-1.07) |
|  | 6+ | - | 589 | 2.54 (1.92-3.36) | - | 1.16 (1.10-1.23) |
| Attitudes towards COVID-19 measures  Reference: About right | Too lenient | - | 423 | 1.07 (0.81-1.41) | - | 1.03 (0.98-1.08) |
|  | Too strict | - | 501 | 0.90 (0.70-1.16) | - | 0.97 (0.92-1.01) |
|  | Don't know | - | 46 | 1.71 (0.75-3.87) | - | 1.08 (0.94-1.23) |
